# An Integrated Multiple Electrochemical miRNA Sensing System Embedded into a Microfluidic Chip

**DOI:** 10.3390/bios12030145

**Published:** 2022-02-27

**Authors:** Pedro Gonzalez-Losada, Martina Freisa, Claire Poujouly, Jean Gamby

**Affiliations:** Centre de Nanosciences et de Nanotechnologies, Université Paris-Saclay, CNRS, 91120 Palaiseau, France; pedro.gonzalez@inl.int (P.G.-L.); martina.freisa@universite-paris-saclay.fr (M.F.); claire.poujouly@universite-paris-saclay.fr (C.P.)

**Keywords:** microfabrication, microfluidics, readout electronics, electrochemical detection, cyclic voltammetry, microRNA

## Abstract

In this article, we present the design, fabrication and characterization of a microfluidic device and a dedicated electronic system to perform 8 multiplexed electrochemical measurements of synthetic miRNA strands, as well as the biochemical protocols developed for the functionalization of the electrodes and the quantification experiments. The outcomes of this work highlight that the parallelization of eight microchannels containing 2-electrode cells driven by the dedicated electronics offers a solution as robust as a conventional 3-electrode cell and commercially available potentiostats. In addition, this solution presents the advantage of simultaneously reduce the microfabrication complexity, as well as offering an integrated; multiplexed and portable system for the quantification of miRNA. The results presented demonstrate that the system shows a linear response on concentrations down to 10^−18^ mol/L of perfect matched reporter and capture sequences of synthetic miRNA.

## 1. Introduction

Micro ribonucleic acids (miRNAs) are a type of noncoding RNA constituted by around 20 nucleotides that are highly involved in post-transcriptional regulation of 30% of the coding genome [1] capable of inducing the extinction or expression of certain genes. Multiple studies have pointed out the relation between their presence in body fluids (plasma, urine, blood, saliva) and different disorders and diseases such as inflammation, cancer or infections, among others [2,3,4,5]. Thus, after their discovery in 1993 [6], over 2000 different types have been identified in humans [7], becoming promising biomarkers due to their rapid, precise, minimally invasive and reliable diagnosis.

Nevertheless, in order to detect the in vivo variation of miRNA expression levels (under femtomolar [8]), a precise, miniaturized, fast and low-cost detection and quantification method still remains a challenge [9]. The traditional miRNA detection methods, such as Northern Blot, microarray sequencing and RT-PCR [10,11,12] are time-consuming, expensive, need particular laboratory instruments (ex. thermocyclers) [10] and, in many cases, do not provide absolute quantification [13]. In addition, the particular characteristics of this nucleic acid molecules such as their shortness and high homology within families, make their detection a challenge for these technologies [14,15,16,17].

Electrochemical sensing has attracted a high interest in recent years due to its inherent sensitivity, specificity, short response time and broad dynamic range [18]. Compared to other methods such as optical sensing (fluorescent techniques) or gravimetric sensing (QCM [19]), electrochemical DNA biosensors present the singular advantage to directly transduce hybridization phenomena into electrical signals that can be easily read by an electronic system. Moreover, these sensors are easily miniaturized and integrated into microfluidic devices, allowing us to take advantage of the microfluidic systems such as low volumes of sample or batch processing [20] and providing portability and compactness [21] in order to easily serve as point of care testing (PoC) systems [13].

In the present study, we introduce a novel electrochemical biosensor platform able to detect and quantify miRNA molecules in the concentration levels of those found in biological samples (femtomolar). In addition to its high sensitivity, the system offers a multiplexed detection of up to eight different miRNAs in the same analysis, as well as a fast, portable and low-cost system that can be used as a point-of-care (POC) system [22].

The proposed system includes a microfluidic device composed of eight channels containing the electrochemical cells functionalized with the capture DNA probes, which constitute the sensing element and readout electronic system capable of performing the parallel and automatized measurements on the different channels.

### 1.1. Electrochemical Cell Embedded into the Microfluidic Device

The electrochemical cell proposed has the singularity of adopting a two-electrode configuration instead of the classical electrochemical configuration composed of three electrodes. The two-electrode setup simplifies the device and results to be more adapted for microliters volume samples [23]. In particular, it uses a microelectrode of 30 µm by 300 µm that acts as a working electrode (WE) and a millimetric electrode of 2 mm by 300 µm for the counter electrode (CE). This second long electrode has also the purpose of maintaining a resting potential stability between the CE and the electrolyte, thus operating also as a pseudo-reference electrode (RE) [23,24,25,26].

The goal of the sensor is the electrochemical detection of the hybridization events of the DNA/miRNA complementary strands on the surface of the WE. The detection techniques used are voltammetric and/or amperometric and consist in the application of a certain voltage that will reduce or oxidize the species of interest on the WE surface. As a result, the electron charge transfer current generated by the faradic reaction is proportional to the concentration of the species of interest [27,28]. These reactions, occurring on the CE surface, generate a counter reaction on the WE surface, and thus a net current flow that is proportional to this charge transfer measured by the electronic circuit.

The electrolyte employed contains equimolar of the 3 mM [Fe^III^(CN)_6_]^3−^/[Fe^II^(CN)_6_]^4−^ redox couple in 0.5 M NaCl. The well-known electrochemical reaction is associated with the one electron transfer. It takes place on the microelectrode during the immobilization and hybridization steps. The equilibrium potential between the two gold electrodes (WE-CE) in this solution is 0 V.

### 1.2. Readout Electronics

The electronic board permits the realization of a so-called cyclic voltammetry (CV) measurement. This electrochemical technique consists in a potential sweep applied between the WE and the CE, and the measurement of the collected current at the WE. For this purpose, the electronic circuit must drive the electrodes with a triangular shaped polarization potential, which produces the reduction and oxidation of the species of interest on the surface of the WE [28,29,30].

Thus, the current generated at the WE—due to the electrons transfer by faradaic processes—is proportional to the DNA/miRNA strands hybridization occurring after the functionalization of the electrodes with the single strand DNA (ssDNA) complementary to the miRNA under investigation. Therefore, measuring the WE current during the CV experiment, it is possible to quantify the miRNA species under investigation in the analyzed sample, obtaining, thus, a direct transduction from the hybridization phenomena to an electric current.

The measurement of this current can be performed following different approaches such as using a shunt resistor in series with the CE and measuring the voltage drop across it [31], or using a so-called switching capacitors configuration where the current flowing through the electrochemical cell is determined by measuring the voltage through a capacitor after a certain time [32].

One of the most common approaches, and the one chosen for our device, is to use a transimpedance amplifier (TIA) stage that directly converts the current flowing through the cell into a voltage. For this, an amplifier containing a resistor (R_TIA_) on its negative feedback loop, is placed in series with the WE. Despite its simplicity, a few factors that may limit its performance must be taken into account during the design phase, such as the input bias current of the amplifier to avoid current leakage, or the thermal and flicker noise introduced by the electronic components. These design considerations will be discussed in the corresponding section.

### 1.3. Sensor Functionalization and Hybridization Protocols

The sensor functionalization protocol permits immobilizing the capture probes—synthetic ssDNA probes complementary to the type of miRNA under detection—on the electrode surface, creating a self-assembled monolayer (SAM).

The techniques used to provide an effective functionalization strictly depend on the electrode material [33]. Although many emerging materials are being explored for electrochemical detection applications, these materials require several conditioning stages before grafting the DNA capture probes, as, for instance, carbon nanotubes (CNT) [34] or graphene [35,36]. For this reason, gold electrodes were used since they provide a simple method to form the self-assembled monolayer (SAM) by the use of thiol-labeled capture probes which present a strong affinity between sulfur and gold [37].

As in the approach adopted by Horny et al. [24], the hybridization is held at room temperature, avoiding thus the integration of heating elements in the microfluidic device. Once the capture probes are immobilized at the gold electrodes, the DNA targets mimicking the miRNA of interest diluted in NaCl are introduced in the microfluidic channel for hybridization with its complementary strands.

## 2. Materials and Methods

### 2.1. Microfluidic Device Microfabrication

**Figure 1:**
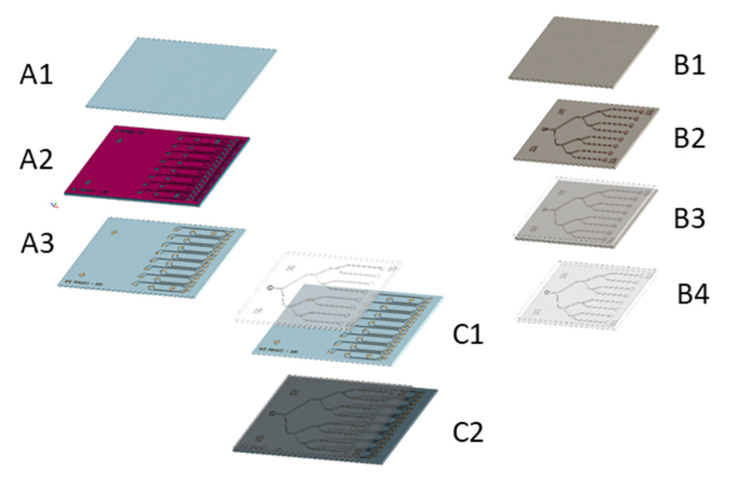
Fabrication process for the microfluidic device realization. (**A1**). Glass substrate. (**A2**). Lithography step. (**A3**). Metal sputtering (50 nm of TiW and 200 nm Au). (**B1**). Silicon substrate. (**B2**). Lithography with 2 µm SU8-2002 and 50 µm SU8-2050. (**B3**). PDMS pouring on silicon mold. (**B4**). PDMS peeling-off. (**C1**,**C2**). PDMS and glass substrate alignment and plasma bonding.

A microfluidic device is formed by two different parts: a glass substrate containing the gold microelectrodes (Figure 1A) and a polydimethylsiloxane (PDMS) cover (Figure 1B) containing the microfluidic channels.

Gold microelectrodes were patterned onto a 4-inch glass wafer (Figure 1(A1)) using a lift-off process. For this purpose, an initial photolithography step was performed (Figure 1(A2)) using a negative photoresist (AZ nLOF2020 from MicroChemicals). Then, a 50 nm layer of TiW was sputtered on the whole wafer to improve the adhesion of the metal to the glass substrate, followed by a 200 nm sputtered layer of Au. The process was completed by removing the photoresist in an acetone ultrasound bath for 15 min, followed by an isopropanol rinse (Figure 1(A3)). Once the electrodes were patterned, the wafer was diced in several chips.

Microchannel structures were fabricated by molding PDMS on a SU8 master mold containing the patterns of the channels. The master mold was fabricated on a 4-inch silicon wafer that acts as substrate (Figure 1(B1)). A 2 µm-thick layer of SU-8 2002 (from MicroChem) was spin coated on the wafer and baked for 2 h at 110 °C to serve as adhesion layer. Then, a 50-micron layer of SU-8 2050 (from MicroChem) was spin coated and the microchannels were patterned by means of optical lithography (Figure 1(B2)). Finally, a mixture of PDMS was prepared mixing 10 parts of silicon elastomer and 1 part of curing agent. This mixture was then poured on the mold (Figure 1(B3)), placed under vacuum during 2 h30 and cured in an oven at 60 °C for 4 h. When the PDMS was polymerized, it was peeledoff from the mold and cut out (Figure 1(B4)).

At the end of the process, the diced electrode chips and the PDMS microchannels were bonded using an oxygen plasma (Figure 1(C1)). The fine alignment of the device is achieved thanks to two different elements: alignment marks and tolerances in the design. The last step consists in being baked at 60 °C for 1 h, resulting in the device shown in Figure 1(C2).

### 2.2. Readout Electronic Circuit

**Figure 2:**
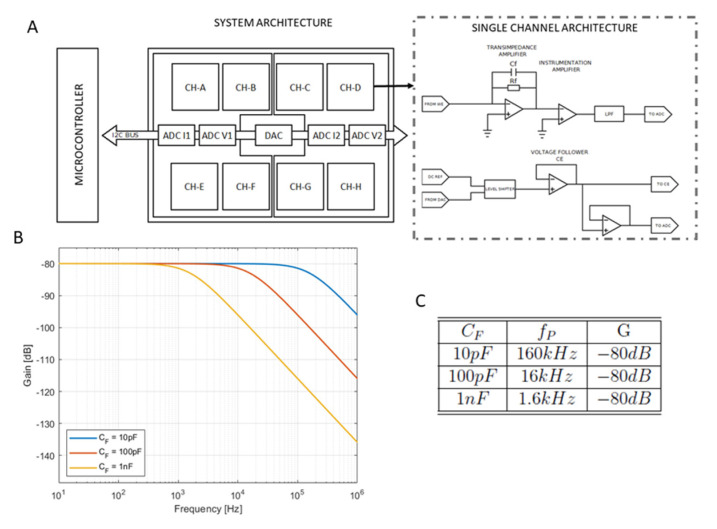
(**A**) Architecture of the 8-channel readout electronics system and schematic of an individual channel. (**B**) Frequency response of the TIA for different capacitor values. (**C**) Summary of the cutoff frequency obtained for the three different capacitors.

The electronic readout circuit is capable of driving up to 8 channels and is composed of 8 individual analog read-out circuits that polarize the electrodes and read the currents and voltages. The polarization signals of the 8 circuits are generated by an 8-channel digital-to-analog converter of 12 bits (AD5629RARUZ-1 from Analog Devices) and the input voltages and currents are sampled by 12 bit analog-to-digital converters (LTC2309 from LinearTechnology, Milpitas, CA, USA), as represented in Figure 2A (system architecture). These components are connected to an Inter-Integrated-Circuit (I^2^C) bus that constitutes the communication channel with the microcontroller of the system. This microcontroller runs the firmware of the system that controls the converters and digital gains as well as the communication with the LabView^®^ interface of the system.

As explained in the introduction, the readout circuit is based on a TIA configuration that permits us to read the output current at the WE, as shown in Figure 2A (single channel architecture), whose output voltage is proportional to the input current, as well as the feedback resistor. In order to increase the stability of the amplifier, a feedback capacitor (*C_F_*) is introduced in parallel to the *TIA* resistor [38,39], introducing a pole in the transfer function of the circuit as written in Equation (1).

(1) $$H\left(j\omega \right)=\frac{R_{TIA}}{1+j\omega C_{F}R_{TIA}}$$

The frequency of this pole is determined by the values of the feedback resistor and the feedback capacitor and expressed as follows:

(2) $$f=\frac{1}{2\pi C_{F}R_{TIA}}$$

Thus, the bandwidth of the *TIA* is limited by the value of these two components and the gain is determined by the feedback resistor. Therefore, a compromise between the amplification of the current and the bandwidth must be found. Considering that the signal we measure is in the range of nanoamperes, a resistor of 100 kΩ provides an output in the range of millivolts that can be amplified in a second stage, keeping the bandwidth of the amplifier at a reasonable value.

Regarding the value of the capacitor, a high value offers better stability but reduces the total bandwidth of the amplifier, prompting us to find a compromise value. For this purpose, simulations were performed on LTSpice to determine the bandwidth of the amplifier when using different capacitors of 10 pF, 100 pF and 1000 pF (Figure 2B).

The required frequency for the TIA stage, considering a maximum scan rate of 50 mV/s, is 1800 Hz. The Bode diagram shows that a value of 10 pF (blue line) offers a higher bandwidth (160 kHz) than the one required. On the other hand, a higher value of 10^3^ pF (yellow line) limits the bandwidth to a few tens of hertz, which is not enough for our requirements. Finally, it can be observed that values around 100 pF (red line) offers a good compromise between bandwidth and stability. Bandwidth-gain values in function of the different capacitor values are summarized in Figure 2C.

### 2.3. Chemical, Sensor Functionalization and Hybridization Protocols

The thiol-labeled capture probes in the channel: the synthetic ssDNA complementary to the corresponding miRNA (miR-122), and the unlabeled DNA target mimicking miR-122 were purchased from Integrated DNA Technologies. The sequences are reported in Table 1. The redox [Fe_(III)_CN)_6_]^3−^/[Fe_(II)_(CN)_6_]^4−^ tracer and sodium chloride salt were obtained from Sigma-Aldrich and used without further purification.

An initial characterization of the microelectrodes, by means of CV, is done in presence of a 0.5 µL/s constant flow rate of 3 mM [Fe_(III)_CN)_6_]^3−^/[Fe_(II)_(CN)_6_]^4−^ in 0.5 M NaCl to characterize the initial state of a gold surface sensor. The flow is monitored by a neMESYS (CETONI GmbH) syringe pump used in all the experiments.

This step is followed by the introduction of the thiol-labeled capture probes in the channel. The probes stay in the microfluidic channels for at least 2 h to ensure a robust SAM formation. Then, 0.5 M NaCl was introduced in the microfluidic channels for 30 min in order to check SAM stability over time. Between each above-mentioned step, an electrochemical characterization of the electrode is performed with the redox tracer (0.5 µL/s constant flow rate of 3 mM Fe^II^/Fe^III^) to determine the formation of the SAM on each electrode surface. The decrease on the measured current confirms that SAM was assembled on the electrode surface, and thus the functionalization process was completed successfully.

Finally, after again rinsing the channels with a 0.5 M NaCl, the DNA targets (complementary sequence reported in Table 1) are introduced in a microchannel. In the reported study, different DNA concentrations are used to test and validate the microfluidic device and the readout electronics. First, the lowest DNA concentration sample is slowly introduced in the microfluidic channel for hybridization. Second, the electrochemical measurement is performed to verify and quantify the hybridization after 30 min (time necessary for the hybridization between complementary strands).

The following list summarizes the different CV characterizations performed during the protocol:

- Bare electrodes, for the characterization of the electrodes;
- Functionalized electrodes, after the SAM formation with the DNA-probes;
- Lowest DNA target concentration, hybridization can be detected in sample containing 10^−18^ mol/L miR-122 concentration;
- Intermediary DNA target concentration, i.e., 10^−12^ mol/L;
- Highest DNA concentration, i.e., 10^−6^ mol/L.

## 3. Results and Discussion

### 3.1. Microfluidic Device Microfabrication

One of the most important elements of the system is the microfluidic device that contains the electrochemical sensors and the channels where the hybridization reactions take place. For its fabrication, we used simple and well-known microfabrication techniques to reduce its complexity and therefore its cost, a required feature for a device conceived to be disposable. Pursuing the goal of reducing the complexity of the system, the 2-electrodes electrochemical cell configuration reduces the number of connections needed and thus the size of the device. Previous works conducted in our group demonstrated the feasibility and advantages of embedding the 2-electrode configuration in the microfluidic device, which lowers the limit of detection down to 1 aM due to a reduction of the diffusion layer and a faster collection of the targets thanks to the forced convection [24].

**Figure 3:**
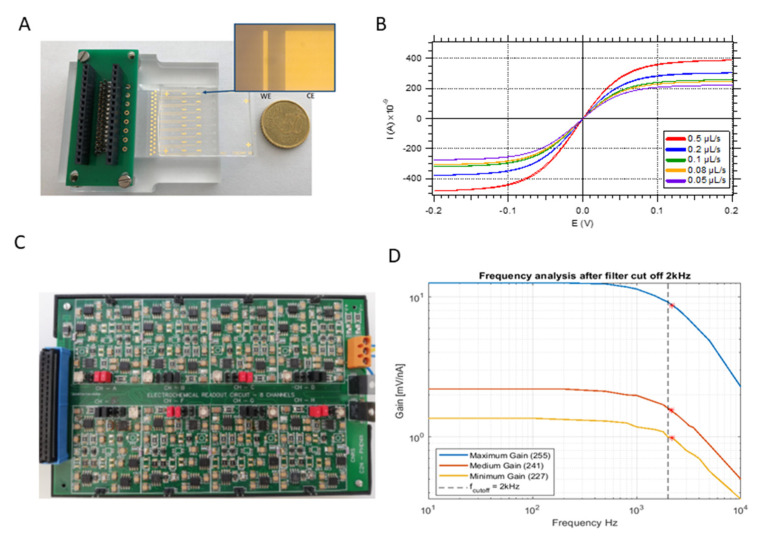
(**A**). Figure representing the final microfluidic device on the chip holder. A zoom on the embedded electrochemical cell is reported. (**B**). Characterization response of the embedded electrochemical cells (using a BioLogic VSP 300) at different flow rates after its fabrication. (**C**). Dedicated electronic circuit developed for the multiplexed readout of up to 8 different sensors. (**D**). Frequency response characterization of the analog stage comprising the current−voltage conversion, amplification and filtering for different values of gain: maximum gain (blue), medium gain (red) and minimum gain (yellow). The dotted vertical line represents the theoretical cut off frequency, and the red stars the found cut off frequency for each measurement.

This simplicity and reduced size allowed us to introduce eight individual channels where eight samples can be analyzed individually and simultaneously (Figure 3A). By functionalizing the 8 channels with different DNA-probes, the chip becomes a multibiomarker tool thanks to its capability to analyze the sample simultaneously in the different channels. The individual functionalization is achieved thanks to the geometry of the microchannels. As shown in Figure 1B, the microfluidic device consists of 1 hole that acts as an inlet of the main channel that successively splits into two branches to achieve 8 parallel channels with one outlet hole per channel. Thus, during the functionalization of the electrodes, the solutions containing the different capture probes are introduced through the individual holes that act as inlets. Then, during the normal operation of the device, the sample is introduced in the common inlet hole and then distributed to the individual channels where the hybridization and electrochemical detection takes place. For now, and in order to validate the system, the same DNA-probe was used for the 8 channels.

The 2-electrodes, working electrode and counter electrode, which compose the electrochemical cell, have a width of 30 µm and 2 mm, respectively, and are separated by 100 µm (see Figure 3A).

The response of the gold electrodes to different flow rates was characterized by showing a direct relation between the levels of current measured and the flow rate on the microchannel, as shown in Figure 3B. The obtained limiting current signature is the one expected in the case of channel-electrode with hydrodynamic flows, as predicted by the Levich’s theory [40] and demonstrated by several authors [41,42,43].

Concerning the geometry of the microchannels, every electrochemical cell is embedded in one individual microchannel that is connected to an individual outlet. The microchannels are interconnected to a single inlet, which allows for distributing the sample in the different microchannels.

### 3.2. Readout Electronic Circuit

A specific circuitry was developed to meet the requirements of the biosensor in terms of type of measurement, measured current levels, number of channels and portability [44,45,46]. Despite that many laboratory potentiostats exist in the market offering very high performances, they are not adapted to our application with regard to cost, portability and number of channels [47]. Other solutions like integrated circuits were also considered and discarded because of the low current level output of our sensor [48]. Finally, some suppliers as Palmsens offer an integrated potentiostat that can perform different electrochemical techniques [49]. However, this solution was not adapted due to the low levels of current of our system (under 100 nA).

In addition, the 2-electrode configuration used for the electrochemical cell simplifies the requirements of the electronic system since no circuitry for the reference electrode (RE) is needed, allowing us to embed 8 different channels able to perform up to 8 parallel electrochemical measurements. The design of the circuit, previously explained, was implemented on a printed circuit board (PCB) that contains the analog circuits to perform the electrochemical measurement, as well as the samplers and communication buses (Figure 3C).

Figure 3D shows the frequency response characterization of the analog stage, comprising different current-voltage conversion, digital controlled amplification and filtering. This characterization was performed by feeding the input of the circuitry with a current source composed of a function generator with a 10 MΩ output resistance that provides a sinusoidal output current of 100 nA. The frequency of the signal was swept from 10 Hz to 10 kHz and the output voltage was measured before the ADC (Analog to Digital converter) to calculate the gain (V_out_/I_in_) of the analog stage. The characterization shows the frequency response for 3 different gain values of the instrumentation amplifier. The experimental cutoff frequencies found are plotted in the curves as red stars, and for the three cases it is around 2.2 kHz. This bandwidth is adapted to the 1.8 kHz maximum required frequency and is consistent with the theoretical value of 2 kHz, defined by the last low pass filter of the system. Finally, Labview interface has been developed to control the instrument and to visualize the multiplexed measurement on the eight different channels.

### 3.3. Sensor Characterization and Functionalization Plus Hybridization Protocols

The experiments carried out to characterize the performance of the system include the use of the electronics, as well as the microfluidic chip containing the electrochemical sensors. The main goal of this section is to show that the whole system is able to detect multiplexed measurements, and, furthermore, the functionalization and hybridization steps. The following subsections report the results obtained for the different experiments.

#### 3.3.1. Simultaneous Measurements over Several Channels

**Figure 4:**
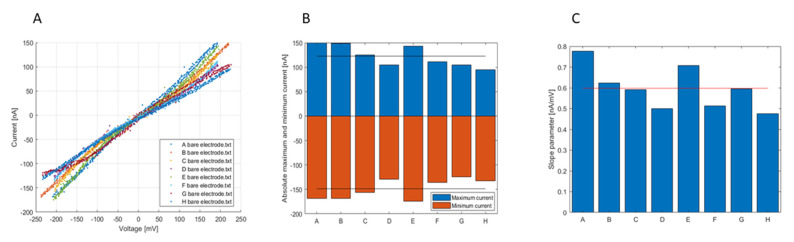
(**A**). CV curves for each channel of the device. Characterization of bare gold electrodes. (**B**) Absolute maximum (blue) and minimum (red) currents histogram for each device channel, values from Figure 4A. The black lines are for the mean values. (**C**) Fitted slope parameters histogram CV curves in Figure 4A, the red line is for the mean value.

The first experiment consisted of simultaneous characterizations of the electrochemical cells embedded in the microfluidic channels. The measurements for each channel are performed synchronously such that it is possible to have the initial characterization for eight bare electrodes. In Figure 4A, the eight CV curves are plotted, the level of the resulted current is proportional to the size of the channel-electrode and the concentration and charge transfer constant of the redox reaction. As expected, it is possible to observe the equilibrium potential at 0 V and the symmetry of the curves.

In order to interpret these results, the stored data was processed with a MatLab^®^ script that divided the curves in three parts and extracts the most important information. These are the maximum zone (plateau: limiting oxidation current), the minimum zone (plateau: limiting reduction current) and the central zone (slope: kinetic current). Maximum and minimum zones are directly extracted from the curves while the central part is linearly fitted, leading to extraction of a slope (m). These parameters are showed in Figure 4B,C. In Figure 4B, the blue histogram is for the maximum and the red one is for the minimum current; the black lines are for the evaluated mean, 122.6 nA and −148.8 nA, respectively. It is possible to observe a few differences in terms of absolute currents among the different bare gold electrodes—with a standard deviation of about 20 nA in both cases—that are probably due to a non-uniform flow of the solution in the different channels and the microfabrication process.

In fact, these discrepancies might be due to the variations on PDMS channel heights that induce small variation in the velocity gradient or the alignment of the PDMS cover on the electrodes that might induce some differences in the size delimited by the PDMS cover on the electrode surface exposed to the electrolyte. Finally, the electronic measurement might induce a small difference between channels due to the discrete character of the gain values, as well as small tolerances of the components.

Analogous results are obtained for the slope parameters (Figure 4C) where the calculated mean slope is 0.6 nA/mV with a standard deviation of 0.1 nA/mV.

Overall, the data shown in this section demonstrates that the electronic system is able to provide Cyclic Voltammetry measurements in a multiplexed way, such that in the future simultaneous miRNA detections can be performed.

#### 3.3.2. Functionalization and Hybridization with Different Target Concentrations

While the detection of the multiple CV curves is a remarkable first validation for our device and system, the real goal is to detect the functionalization and hybridization events at different concentrations. For this purpose, the experiments were carried out with different concentrations down to 10^−18^ mol/L, which is compatible with our limit of detection defined by working electrode sizes.

In this subsection, the results for the functionalization and hybridization with different reporter probes concentrations are reported. These measurements are performed according to the functionalization and hybridization protocol explained in Section 2.3 using the following concentrations: 10^−18^ mol/L is defined as minimum risk, 10^−12^ mol/L is defined as medium risk and 10^−6^ mol/L is defined as maximum risk.

**Figure 5:**
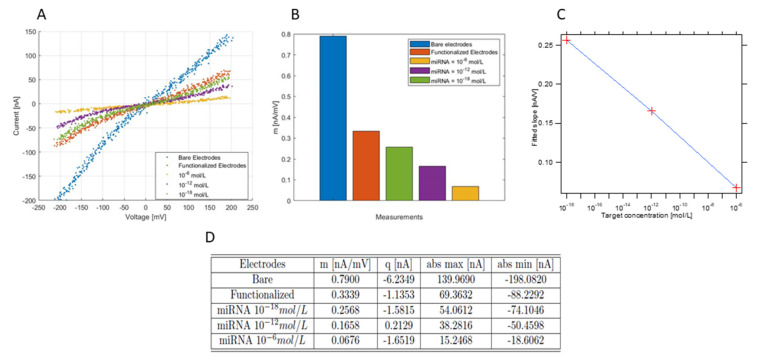
(**A**) Example of CV experiments for the hybridization in channel A (see Figure 4B). Blue curve for the bare electrodes, red curve for functionalized electrodes with ssDNA probes, green curve for a miRNA target concentration of 10^−18^ mol/L, purple curve for a miRNA target concentration of 10^−12^ mol/L, yellow curve for a miRNA target concentration of 10^−6^ mol/L. (**B**) Histogram representing the extracted slope by MatLab for each CV curve in Figure 5A. (**C**) Graph representing the three slopes for the hybridization process respect to target concentration in log scale representation. (**D**). Table for the five measurements in which the important values are extracted by an implemented MatLab code. The first column, *m*, represents the slope parameter, *q* the intercept of the fit, *abs max* the CV curve absolute maximum and *abs min* the CV curve absolute minimum.

Figure 5A shows the voltammograms of the microelectrode measured in the presence redox tracer under flow rate, as defined above at different stages of the detection protocol: bare gold electrode (blue dotted curve), after functionalization (red dotted curve), after hybridization with the lowest concentration (green dotted line), the middle concentration (violet dotted curve) and the highest concentration (yellow dotted curve). These voltammograms show a decrease of the absolute current values due, first to the SAM formation after the functionalization of the microelectrode, and second to the following hybridization of the target probes at different concentration levels.

In order to extract the information from these voltammograms, they were treated with a MatLab^®^ script to obtain their slopes, as previously described. Results are shown in Figure 5B, where the extracted slope of the different curves is shown in a histogram. This histogram clearly shows a decrease in the slope of the voltammograms: 0.790 nA/mV for the bare gold electrode, 0.334 nA/mV for the functionalized electrode, 0.257 nA/mV for the hybridization with the minimum concentration, 0.166 nA/mV for the hybridization with the medium concentration and 0.0676 nA/mV for the hybridization with the maximum concentration (Figure 5D).

To summarize, the slope of the measured curve is related to the faradic current of the electrochemical cell that is directly related to the area of the electrode exposed to the redox tracer solution. When the electrodes are not functionalized, the gold surface of the electrode is completely exposed to the solution, and, thus, the faradaic current is maximum. After the formation of the self-assembled monolayer (SAM) on the surface of the electrodes, the coverage of the electrode increases, and, thus, the faradaic current and the slope reduces. With higher concentrations of miRNA, the electrode surface coverage due to the hybridization between the immobilized DNA-probes with the DNA targets increases, and, thus, the slope decreases.

Data from this histogram allowed us to trace the calibration curve of the sensor (Figure 5C) where the slope of the different voltammograms are shown for the different concentrations in a logarithmic scale representation. Linear fitting of this curve allowed us to extract the slope (1.577 × 10^−2^ (nA/mV)/(log (mol/L))) and the offset (1.0033 × 10^−2^ nA/mV). Taking into account the logarithmic scale of the concentration, we can write the Equation (3) that links the slope of the voltammograms (in nA/mV) and the concentration of the sample as follows:

(3) $$\mathrm{Log}\left(C\right)=-1.577\times 10^{-2}\times VS-1.00\times 10^{-2}$$

where *C* is the concentration and *VS* represents the slope of the voltammogram.

Therefore, the concentration in mol/L as function of the slope of the measured voltammogram is defined in Equation (4) as follows:

(4) $$C=10^{\frac{-\left(VS+2.58*10^{-2}\right)}{1.577*10^{-2}}}$$

This Equation (4) allows us to obtain the concentration of the sample by extracting the slope of the voltammogram.

To conclude, the limitation for higher target concentrations (here 10^−6^ mol/L) are determined by the saturation of the built SAM. For instance, if the surfacic concentration of the probes is too high, the hybridization with its complementary targets will be difficult due to the steric hindrance on the SAM. Thus, the working concentration between 10^−6^ and 10^−7^ mol/L used for SAM construction is optimal to promote the hybridization step instead of a range between 10^−5^ and 10^−4^ mol/L where the surface sensor behaves as blocking electrodes. Concerning the minimum value explored in this work (10^−18^ mol/L), the levels of current associated with lower concentrations would be under nA, so the characteristics of the electronic system might need further development to adapt them and keep the performances shown in the range of concentrations presented in this work.

## 4. Conclusions

In this work, the whole chain of the instrument development is presented: the conception and fabrication of the microfluidic chip containing eight channels for DNA sensing, the development of the multiplexed readout electronics and the validation of the different steps of the biochemical protocols (functionalization and hybridization) using both elements. The employed synthetic biological samples are perfectly matched reporter and capture sequences that demonstrate the capabilities of the system to provide a linear response on concentrations down to 10^−18^ mol/L. In the future, sequences differing on one or several nucleobases will be used to verify the influence of these mismatches in the electrical response of the system and its detection. In addition, biological in vivo samples should be pre-treated with commercially available miRNA extraction kits and then used in the device to assess the performance of the whole system in in vivo conditions [46].

## Figures and Tables

**Table 1:** DNA probes and DNA targets base sequencing mimicking the miRNA 122.

Oligonucleotide	Sequence (5′ to 3′)
DNA capture Probe	5′-thiol C6-CAA ACA CCA TTG TCA CAC TGC-3′
miRNA Target	5′-GC AGT GTG ACA ATG GTG TTT G-3′

## Data Availability

Data sharing is not applicable to this article.
